# New Operation for Inverted Uterus

**Published:** 1870-08

**Authors:** 


New Operation for Inverted Uterus.—Dr. T. G. Thomas gives an account of four cases of chronic inversion of the uterus, with a description of a new operation for its relief.

After giving a history of the accident and its remedy, as formerly treated, he quotes from a foreign journal a resume of fifty-eight cases operated upon for the removal of the inverted uterus by ligatures, knife, and ecraseur. Eighteen terminated fatally, being nearly one-third of all operated upon.

The new operation, which is to be used only after all other means have failed, he has performed successfully upon a patient who had submitted to fourteen attempts to reduce by taxis, each, with on eexception, lasting more than an hour. The operation consists in opening the abdominal cavity by a brief incision through the median line just above the uterus, passing a pair of dilating forceps into the cavity of the cervix and dilating it, then by pressure in the vagina upon the fundus of the organ returning it to its place. The dilation was easy and rapid, but on withdrawing the dilator the issue of the organ again contracted, making a more prolonged dilatation necessary. There was, by the great pressure upon the fundus of the uterus, a small rent made between that organ and the bladder. After the operation there was profuse hemorrhage from an artery cut in an operation the day before, but against these unfavorable circumstances, and that of the abdominal wound, the patient made a rapid recovery.

This operation seems like a bold procedure, but that this is an error we need only to reflect upon the comparative safety in modern times of the explorative incision for ovariotomy, and that of all operations of amputation of the inverted uterus, one-fourth to one-third result fatally.

Dr. T. says that in a recent case he would use belladonna per enema and the warm douche to the uterus, kept up for a week, so as to relax the uterine tissue as far as possible, then for another week he would employ pressure to the uterus by means of a caoutchouc bag filled with air or water. After this he would employ taxis for an hour or more once or twice a week, in the mean time keeping up vaginal pressure by the caoutchouc bag. Having failed with these means, and not before he would resort to the abdominal section, modifying the operation by using a dilator with four limbs instead of two, and keeping the instrument in place until the tendency to contract again was somewhat worn out.

Of the danger of rupturing the vagina by pressure against the fundus, he says: "I should prefer to trust a patient to the operation of abdominal section than to that of prolonged taxis at the hands of a rough, unintelligent, and inexperienced practitioner.—*New York Med. Journal.*

